# Assessment of genetic polymorphisms associated with malaria antifolate resistance among the population of Libreville, Gabon

**DOI:** 10.1186/s12936-023-04615-1

**Published:** 2023-06-14

**Authors:** Sylvatrie-Danne Dinzouna-Boutamba, Berthe Amélie Iroungou, Falone Larissa Akombi, Lauriane Yacka-Mouele, Zin Moon, Ja Moon Aung, Sanghyun Lee, Dong-Il Chung, Yeonchul Hong, Youn-Kyoung Goo

**Affiliations:** 1 Department of Parasitology and Tropical Medicine, School of Medicine, Kyungpook National University, Daegu, 41944 Korea; 2 Unité Mixte de Recherche Centre International de Recherches Médicales de Franceville et le Service de Santé Militaire, Libreville, 20404 Gabon; 3 Division of Bio Bigdata, Department of Precision Medicine, Korea National Institute of Health, Korea Disease Control and Prevention Agency, Cheongju, 28159 Korea

**Keywords:** Malaria, Mutation, Haplotypes, Artemisinin combination therapy, Resistance, Libreville

## Abstract

**Background:**

Gabon is a malaria-threatened country with a stable and hyperendemic transmission of *Plasmodium falciparum* monoinfection. Malaria drug resistance is widely spread in many endemic countries around the world, including Gabon. The molecular surveillance of drug resistance to antifolates and artemisinin-based combination therapy (ACT) is one of the strategies for combating malaria. As *Plasmodium* parasites continue to develop resistance to currently available anti-malarial drugs, this study evaluated the frequency of the polymorphisms and genetic diversity associated with this phenomenon among the parasites isolates in Gabon.

**Methods:**

To assess the spread of resistant haplotypes among the malaria-infected population of Libreville, single nucleotide polymorphisms linked to sulfadoxine–pyrimethamine (SP) and artemisinin drugs resistance were screened for *P. falciparum dihydrofolate reductase* (*Pfdhfr*), *P. falciparum dihydropteroate synthase* (*Pfdhps*), and *P. falciparum kelch 13-propeller domain* (*Pfk13*) point mutations.

**Results:**

The analysis of 70 malaria-positive patient samples screened for polymorphism showed 92.65% *(n* = 63) mutants vs. 7.35% (*n* = 5) wild parasite population in *Pfdhfr*, with high prevalence mutations at S_108_N(88.24%, *n* = 60), N_51_I(85.29%, *n* = 58), C_59_R(79.41%, *n* = 54); however, I_164_L(2.94%, *n* = 2) showed low frequency mutation. No wild haplotype existed for *Pfdhps*, and there were no mutations at the K_540_E, A_581_G, and A_613_T/S positions. However, the mutation rate at A_437_G(93.38%, *n* = 62) was the highest, followed by S_436_A/F(15.38%, *n* = 10). A higher frequency of quadruple IRNI–SGKAA (69.84%) than quintuple IRNI–(A/F)GKAA (7.94%) mutations was observed in the *Pfdhfr*–*Pfdhps* combination. Furthermore, none of the mutations associated with ACT resistance, especially those commonly found in Africa, were observed in *Pfk13*.

**Conclusions:**

High polymorphism frequencies of *Pfdhfr* and *Pfdhps* genes were observed, with alternative alanine/phenylalanine mutation at S_436_A/F (7.69%, *n* = 5) for the first time. Similar to that of other areas of the country, the patterns of multiple polymorphisms were consistent with selection owing to drug pressure. Although there was no evidence of a medication failure haplotype in the studied population, ACT drug efficacy should be regularly monitored in Libreville, Gabon.

**Supplementary Information:**

The online version contains supplementary material available at 10.1186/s12936-023-04615-1.

## Background

Malaria remains one of the most infectious and deadly diseases worldwide, caused by the *Plasmodium* parasite. The World Health Organization (WHO) reported about 247 million cases from 84 malaria endemic countries and 619,000 estimated malaria deaths in 2021 [1]. Most malaria deaths were reported in the WHO African region, with almost 76% of the total deaths recorded in children under 5 years old [1, 2]. In addition to the four African countries accounting for the highest malaria rates, malaria epidemiology in Gabon, a country in Central Africa, is characterized by a stable and hyperendemic transmission of > 90% *Plasmodium falciparum* monoinfection along with mixed infection of various species in a single individual [1–4]. In endemic areas, the most vulnerable target population for malaria disease includes children and pregnant women [1, 5, 6]. However, the malaria burden persists among residents of perennial transmission zones, where asymptomatic carriers are reportedly vast parasite reservoirs and are often adults because of acquired immunity within the exposure time and age [7].

Malaria antifolate drug resistance in the population of Gabon was previously described in 1995, which has gradually increased with the persistent use of these drugs [8]. Following the official implementation of artemisinin-based combination therapy (ACT) under the WHO’s recommendation to reduce the risk of drug resistance in 2003, the first-line treatment for nonsevere *P. falciparum* malaria has been artesunate + amodiaquine (AS + AQ) or artemether–lumefantrine (AL), and sulfadoxine–pyrimethamine (SP) has been the intermittent preventive treatment (IPT) for pregnant women [6, 9, 10]. Moreover, various studies have evidenced that the drug failure of SP is linked to point mutations, including N_51_I, C_59_R, S_108_N, and I_164_L in *dihydrofolate reductase* (*dhfr*) and S_436_A**/**F, A_437_G, K_540_E, A_581_G, and A_613_T**/**S in *dihydropteroate synthase* (*dhps*) [11–13]. The combined quintuple mutation (I_51_R_59_N_108_G_437_E_540_) in the *Plasmodium* parasite has been strongly associated with reduced parasite clearance ability, and SP showed reduced efficacy as an IPT in pregnancy [12, 14]. Since the introduction of these artemisinin-based drug combinations, polymorphisms in *dhfr* and *dhps*, which are linked to their resistance, have been continuously spreading across the nation in different regions [15–18].

Recently, mutations at the Kelch 13-propeller domain were identified to play a key role in delaying parasite clearance following ACT, especially at the C_580_Y, Y_493_H, R_539_T, and I_543_T locations, in a Southeast Asia population [19, 20]. Several studies conducted in Africa did not report these specific Asian *Pfk13* mutations strongly associated with artemisinin resistance [21–24]. However, patients carried other common non-synonymous mutations, such as A_578_S [21, 25, 26]. These observations raise real concerns regarding the efficacy of the drugs used in combination [27, 28]. Nevertheless, *Pfk13* mutation-associated artemisinin resistance in the propeller domain of the parasites is yet to be confirmed among the field samples in Gabon [18, 29]. Therefore, this study aimed to (i) screen the status of circulating *dhfr*, *dhps*, and *K13* haplotypes according to the polymorphisms associated with SP and ACT resistance and (ii) estimate the frequency of the mutations (single to quintuple) of each gene and in the combination *dhfr*–*dhps* genes in the *Plasmodium* parasites obtained from the population in Libreville, Gabon.

## Methods

### Study area and study design

**Fig. 1:**
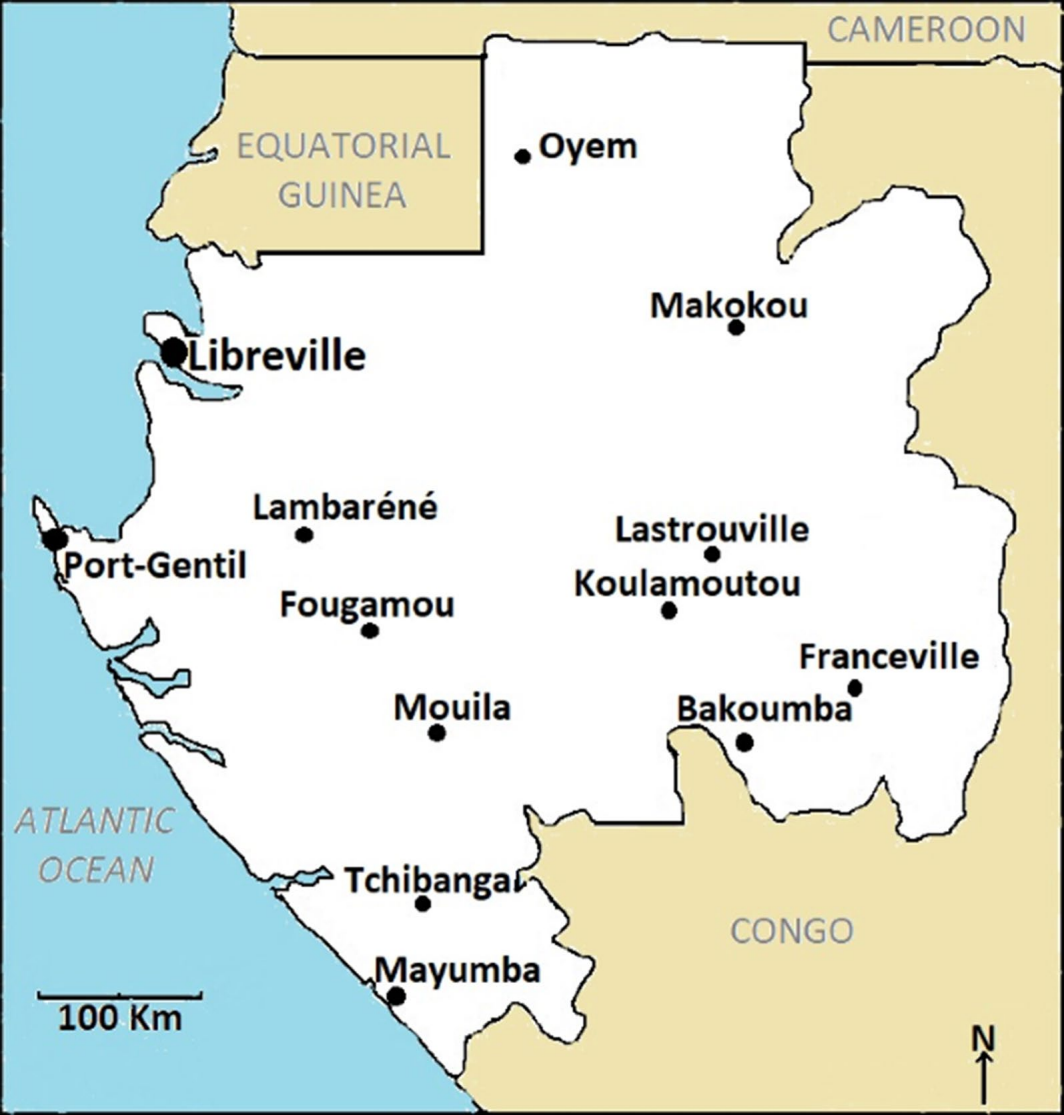
Localization of the study site, Libreville in Gabon

Gabon is located in the sub-Saharan region along the Atlantic coast of Central Africa where malaria is endemic throughout the country. Libreville is the capital city of Gabon and home to one third of the country's population, located in the northwestern province of Estuaire bordering the Komo River (Fig. 1).

In Gabon, malaria is one of the leading causes of consultation and hospitalization, and most *P. falciparum* prevalence statistics are obtained from clinical data [10, 30]. Over the last couple of decades, fluctuations in the decrease or increase of malaria prevalence reported in Libreville’s urban area were 24–42% [30–33]. Moreover, there is an increased prevalence of *P. falciparum* asymptomatic carriage in the population and a scarcity of documentation [30, 32, 34, 35]. This study included asymptomatic and symptomatic patients who were thought to be potential carriers of malaria parasites. From the total number of malaria-confirmed carriers, only 70 samples were included in the resistance profile study. However, the final sample size (N) for each gene marker investigated was determined by considering the total number of observations obtained through sequencing and validating them twice. Thus, the frequency distribution of a haplotype was calculated by dividing the frequency of each occurrence (n) by the total number of observations (N) and multiplying by 100 to obtain a percentage value.

### Study population and ethics approval

From June to July 2019, patients attending the Army Hospital (HIABO) who presented with or without malaria-related symptoms, including fever, general body weakness, chills, and headache, and who were asked by the physicians to take a malaria test were randomly enrolled according to the regulations of the ethics committee. Each patient or caregiver of the child was asked to complete and sign a written consent form before the recruitment. Thus, the study population included 94 women and 74 men between the ages of 5 months and 81 years. The study protocol was reviewed and approved by the National Ethics Committee of Gabon (PROT N°030/2018/PR/SG/CNE).

### Laboratory procedures for malaria diagnosis

A rapid diagnostic test was performed using ABON™ Plus Malaria Pf/Pan Rapid Test Device (Biopharm, China), and regardless of the RDT results, blood samples of the patients were collected using dry Whatman filter paper. The spotted blood samples were dried, and each sample was placed in a single desiccant sachet for storage. Genomic DNA was extracted from the dried blood spots using DNeasy® Blood & Tissue Kit (QIAGEN, Germany) according to the manufacturer’s instructions. To confirm whether the patients had a monoinfection or multi-infection of the *Plasmodium* species, all the samples were amplified via conventional polymerase chain reaction (PCR) assays targeting the small subunit ribosomal RNA genes (*18s*) according to Singh’s protocol [36]. Only the samples showing monoinfection with *P. falciparum* were considered for the analysis of the resistance markers.

### Detection of ***Pfdhfr***, ***Pfdhps***, and ***Pfk13***mutations via nested PCR assay

In agreement with previous studies [19, 37–40], the protocols for evaluating the potential point mutations that induce anti-malarial drug resistance in *Pfdhfr*, *Pfdhps*, and *Pfk13* were slightly modified. The primer pairs used for the primary and secondary PCR amplifications are listed in Additional file 1: Table S1. The mutations examined in the current study are as follows; four mutations at the codons N_51_I, C_59_R, S_108_N, and I_164_L for *Pfdhfr*, five mutations at the codons S_436_A/F, A_437_G, K_540_E, A_581_G, and A_613_T/S for *Pfdhps*, and six mutations at the codons Y_493_H, R_539_T, I_543_T, A_578_S, C_580_Y, and V_589_I for *Pfk13*.

Both primary and secondary PCR were conducted with 50 µL reaction volume containing ~ 25 ng genomic DNA, and 0.5 µL primary PCR products was used as the DNA template for the secondary reaction. All the amplification reactions were conducted using Biometra Tone 96 thermal cycler under the following conditions. For *Pfdhfr*, both amplification reactions were initiated with denaturation at 94 °C for 3 min followed by 40 cycles of denaturation, annealing, and extension at 94 °C/1 min, 48 °C/2 min, and 72 °C/1 min, respectively, with a final extension at 72 °C/10 min for the primary PCR. Furthermore, secondary PCR involved 94 °C/30s, 50 °C/20s, and 72 °C/1 min, with 5 min of final extension at 72 °C. The cycling conditions for *Pfdhps* were the same, except the annealing temperatures of 52 °C/2 min and 53 °C/20s for the primary and secondary PCR amplifications, respectively. However, *PfK13* cycling was performed as described by Ariey et al. [19]. The purified genomic DNA from *P. falciparum* 3D7 clone and distilled water were used as positive and negative controls, respectively. The purified PCR amplicons were sent for sequencing to Macrogen (Korea) where Sanger sequencing of the reaction products was performed using ABI PRISM 3730XL Analyzer (96 capillary type). Further, the obtained sequences data were analysed using Clustal Omega for multiple sequence alignment (https://www.ebi.ac.uk).

## Results

### Distribution of polymorphisms in dhfr, dhps, and kelch 13

Of the 168 samples, only 43.45% (*n* = 73) were positive for *P. falciparum* infection as determined via PCR targeting *18sRNA*. However, because of the very poor DNA quality of 3 samples, 70 positive samples were preserved to analyse the resistance profile harboured among them. Among the 70 *P. falciparum* isolates, 68, 65, and 58 samples were successfully sequenced for *Pfdhfr*, *Pfdhps*, and *Pfk13*, respectively. After comparing the major codons mainly responsible for inducing anti-malarial drug resistance, the frequency results of the mutant alleles were obtained (Table 1). Percentages of *Pfdhfr* mutations were detected in 88.24% (*n* = 60) for the codon S_108_N, 85.29% (*n* = 58) for the codon N_51_I, 79.41% (*n* = 54) for the codon C_59_R, and 2.94% (*n* = 2) for the codon I_164_L. Mutations were not identified at the codons K_540_E, A_581_G, and A_613_T/S in *Pfdhps*. However, mutant alleles were detected in 93.38% (*n* = 62) for the codon A_437_G and 15.38% (*n* = 10) for the codon S_436_A/F in *Pfdhps*. Moreover, none of the known mutations for *Pfk13* that induce artemisinin resistance were observed in the analysed samples (*n* = 58).

**Table 1:** Frequency distribution of *Pfdhfr*, *Pfdhps*, and *PfK13* point mutations vs. wild-type in *P. falciparum* isolates from Libreville

Genes	Codon	Wild-type % (*n*)	Mutated % (*n*)
*dhfr* (*N* = 68)	S108**N**	11.76 (8)	88.24 (60)
	N51**I**	14.71 (10)	85.29 (58)
	C59**R**	20.59 (14)	79.41 (54)
	I164**L**	97.06 (66)	2.94 (2)
*dhps* (*N* = 65)	S436 (**A/F**)	84.62 (55)	15.38 (10)
	A437**G**	4.62 (3)	93.38 (62)
	K540**E**	100 (65)	0 (0)
	A581**G**	100 (65)	0 (0)
	A613**S**	100 (65)	0 (0)
*k13* (*N* = 58)	Y493**H**	100 (58)	0 (0)
	R539**T**	100 (58)	0 (0)
	I543**T**	100 (58)	0 (0)
	A578**S**	100 (58)	0 (0)
	C580**Y**	100 (58)	0 (0)
	V589**I**	100 (58)	0 (0)
	E/G 605** K**	100 (58)	0 (0)

Mutant alleles are in bold and underlined, *N*: total sample size, *n*: sample size.

### Prevalence of ***Pfdhfr***, ***Pfdhps***, and***Pfdhfr–Pfdhps ***combined haplotypes

To determine the distribution of multiple mutations in the population of Libreville, haplotype analysis was performed (Table 2). *Pfdhfr* revealed a high mutation rate (92.65%, *n* = 63) against the wild-type parasites (7.35%, *n* = 5). Triple mutations were more frequently detected (77.95%, *n* = 53) than double mutations and single mutations, which were equally detected (7.35%, *n* = 5). The predominant IRN haplotype (76.48%, *n* = 52), with the N_51_I, C_59_R, and S_108_N codons was responsible for this finding. There was no wild-type for *Pfdhps* among the genotyped samples, and the single mutation was more predominant (89.25%, *n* = 58) than the double mutation (10.76%, *n* = 7). SGKAA was the highest single mutant haplotype (84.62%, *n* = 55) mainly due to the A_437_G mutation point. The analysis of the overall mutation prevalence of the combined *Pfdhfr–Pfdhps* genotypes included 63 fully sequenced samples, identifying 13 haplotypes. The quadruple mutation (69.84%, *n* = 44) was present in majority of the isolates, followed by double, triple, and quintuple mutations that were equivalent to 7.94% (*n* = 5) and single mutations (6.35%, *n* = 4). The common quadruple genotype was primarily from the combination of the N_51_I, C_59_R, S_108_N, and A_437_G mutant genotypes (63.49%, *n* = 40), whereas the quintuple haplotype combined the N_51_I, C_59_R, S_108_N–S_436_A/F, and A_437_G mutations (7.94%, *n* = 5).

**Table 2:** Frequency of haplotypes of *Pfdhfr*, *Pfdhps*, and combined *Pfdhfr–Pfdhps* in *P. falciparum* isolates

Gene	Types	Haplotype	Frequency^a^ (*n*)
***Pfdhfr*** *(N = 68)*	Wild	NCSI	7.35 (5)
	Single mutation	**I**CSI	1.47 (1)
		NC**N**I	5.88 (4)
	Double mutation	**IR**SI	1.47 (1)
		**I**C**N**I	4.41 (3)
		**I**CN**L**	1.47 (1)
	Triple mutation	**IRN**I	76.48 (52)
		N**RNL**	1.47 (1)
***Pfdhps*** *(N = 65)*	Wild	SASKAA	0 (0)
	Single mutation	**A**AKAA	4.62 (3)
		S**G**KAA	84.62 (55)
	Double mutation	(**A**/**F**)**G**KAA	10.76 (7)
***Pfdhfr*** – ***Pfdhps*** *(N = 63)*	Wild	NCSI–SASKAA	0 (0)
	Single mutation	NCSI–S**G**KAA	6.35 (4)
	Double mutation	**I**CSI–S**G**KAA	1.59 (1)
		NC**N**I–S**G**KAA	4.76 (3)
		NCSI–**FG**KAA	1.59 (1)
	Triple mutation	NC**N**I–**FG**KAA	1.59 (1)
		**I**C**N**I–S**G**KAA	3.17 (2)
		**I**CS**L**–S**G**KAA	1.59 (1)
		**IR**SI–S**G**KAA	1.59 (1)
	Quadruple mutation	**IRN**I–S**G**KAA	63.49 (40)
		**IRN**I–**A**AKAA	4.76 (3)
		N**RNL**–S**G**KAA	1.59 (1)
	Quintuple mutation	**IRN**I–**AG**KAA	3.17 (2)
		**IRN**I–**FG**KAA	4.76 (3)

Mutant alleles are in bold and underlined, *N*: total sample size, *n*: sample size, ^**a**^Frequency in percentage

## Short trends review of the resistance

Monitoring drug resistance over time remains crucial for controlling malaria. To assess the constant spread of resistant haplotypes and evaluate its trends, the polymorphism patterns observed in the study population (100%, *n* = 63) were compared with those reported in previous studies (Table 3). The studied population in the Bakoumba city of Gabon harbored ~ 64.3% IRN-G (quadruple) vs. 1.23% IRN-AG (quintuple) mutations before ACT implementation [41]. Following the free use of ACT among the population, compared with the quadruple mutations, the quintuple mutations were almost absent between 2005 and 2014 in certain studied areas, such as Oyem [42], Koulamoutou, Lastrouville, and Franceville [18]. However, quintuple sets of mutations were found in 2005 in Lambaréné (8%) and Libreville (17.9–22%) [15, 17, 43]. For years, malaria parasites have constantly maintained high prevalence rates of multiple sets of quadruple mutations [42–45]. The data showed that the quintuple mutation rates increased while a wide range of single nucleotide polymorphism (SNP) point mutations also simultaneously increased [41–43, 45].

**Table 3:** Short literature review of the distribution of the combined *dhfr/dhps* mutations in *P. falciparum* in the population of Gabon after introducing ACT

Year of collection	Quadruple^a^ (%)	Quintuple^b^ (%)	Sites of collection	References
2000	64.3	1.23	Bakoumba	[18]
2005	34.5	0	Oyem	[45]
2005	69.8	17.9	Libreville	[37]
2005–2006	53	22	Libreville	[6]
2005–2007	59	8	Lambaréné	[17]
2008	64.8	0	Oyem	[45]
2011	100	0	Franceville	[18]
2011	79.7	75.6	Libreville	[37]
2013	100	0	Franceville	[18]
2013	83.3	0	Lastrouville	[18]
2013	73.3	0	Koulamoutou	[18]
2014	88.9	0	Franceville	[18]
2014	100	0	Lastrouville	[18]
2014	91.7	0	Koulamoutou	[18]
2016	93.1	62.8	Fougamou	[47]
2019	69.84	7.94	Libreville	Present data

^**a**^ Quadruple mutation with IRN-G as the major haplotype,^**b**^quintuple mutation including IRN-AG and others haplotypes

## Discussion

The trend of malaria transmission in Libreville, the capital city of Gabon, exhibits an extensively dynamic pattern throughout the rainy season and high heterogeneity according to district areas [46]. Since approximately two decades, SP administration has been limited to intermittent preventive treatment in pregnancy (IPTp)-SPs for pregnant women in Gabon, and a few studies have been conducted on pregnant women between 2005 and 2006 and 2005 vs. 2011 to determine the levels of SP-based ACT drug resistance in the population of Libreville [6, 15, 43, 47]. Additionally, a 2015–2016 study involving the adult population reported a combination of data associated with the cross resistance of SP and trimethoprim–sulfamethoxazole (cotrimazole, CTX) drugs in Oyem and Koulamoutou, including Libreville [34]. The molecular basis of the resistance of the parasites against the antifolates drugs has been clearly associated with mutations in the *dhfr* and *dhps* genes of *P. falciparum* [11]. Following the introduction of antifolates drugs and implementation of ACT in Gabon, a notable rise in parasite polymorphisms has been reported [15, 16]. Consequently, the current prevalence of these drug-resistant haplotypes among the population of Libreville, Gabon was investigated.

In vitro and in vivo studies reported that the S_108_N mutation in *Pfdhfr* was sufficient to confer low-level resistance toward pyrimethamine. Additive mutations at N_51_I and C_59_R caused high-level resistance toward the latter drug in combination with the mutation at I_164_L [17, 41]. The present study revealed high mutations rates at these positions (Table 1), which induce a moderate-to-high level resistance toward pyrimethamine. During the early use of antifolates in Gabon, the *Pfdhfr* triple IRN mutation rates were low before the year 2000 at Franceville and Lambaréné (13.58% and 34%, respectively) [8, 16]. A drastic increase in the mutation rates of up to 100% was observed between 2011 and 2013 at Franceville [18]. Although no wild phenotype was observed among the Lambaréné, Koulamoutou, and Franceville populations in 2014 [18], only 7.35% of the wild-type phenotype was detected among the isolates in the present investigation, which was close to that of the population of Fougamou [45]. However, the I_164_L mutation was detected in two isolates (2.94%), and no mutations were detected in the *Pfdhfr* codons A_16_V and C_50_R nor the codon S_108_T, which is associated with cycloguanil resistance, in the present study [16, 41]. Since the deployment of SP, a high incidence of triple mutations has been reported across the country [15, 17, 18, 34], with the triple mutant IRNI haplotype (76.48%) maintaining dominance over the single and double mutations of the studied population (7.35% each) over the years, as shown in this present study. This was consistent with earlier findings at Bakoumba (72%) [41], Oyem (72–92%) [42], and Lambaréné (92%) [17]. Similar to previously reported high levels of resistance across the country, these resistance patterns occurred because the selected parasites are constantly spreading under the influence of drugs.

Similar patterns involving high triple mutant prevalence were observed in the population of other African countries, such as Cameroon, Congo, Equatorial Guinea, Senegal, and Tanzania [23, 44, 48–50]. On comparing with the high multiple mutations sets documented in Nigeria [51] and Kenya [52], where various SNPs increased the double, triple, and quadruple mutation sets in *Pfdhfr*, likewise NRNL, IRSI, ICNI, and ICNL multiple mutation sets were expressed among the current studied population. In addition, contrary to the I_164_U finding in individuals with malaria infection living with human immunodeficiency virus, I_164_L mutation, which is rarely reported in Central Africa [34, 49, 53] and somewhat reported in East and South Africa and Asia [54–56], was detected in two isolates of the Gabon population of the current study. Thus, these findings indicate the importance of regularly checking the status of the polymorphisms that induce drug resistance.

Regarding *Pfdhps*, no wild phenotype was detected in the studied population. Notably, after the 2005–2007 period, *Pfdhps* wild-type was not detected in the screened populations [16–18, 45], and similarly, a shift from the high selection of the S_436_A mutation to that of the A_437_G mutation following the full implementation of IPTs-SP and ACT was reported [8, 16–18]. Herein, the frequency of the A_437_G single mutation was higher than that of the alternative alanine/phenylalanine mutation (S_436_ (A**/**F)), as shown in the results (93.38% vs. 15.38%, respectively). In contrast to the findings of the isolates from Kenya, the present results did not detect S_436_H mutation [52], instead S_436_F mutation was detected for the first time in five samples among the current studied population of Gabon, similar to that in the population of other countries, such as Sierra Leone, Kenya, and Vietnam [11, 57]. Moreover, it was particularly detected as a FGKAA double mutation (10.67%) (Table 2) in the current study population, whereas no triple or quadruple mutations were observed in *dhps*.

Herein, *Pfdhfr* and *Pfdhps* mutations were commonly identified as multiple mutations than single mutations in the isolates. However, within 10 years following the introduction of ACT and IPTp–SP and its daily use as prescription drugs, a number of investigations highlighted two- to three-fold rates of multiple SNPs among the parasite population owing to high levels of triple and quadruple mutations. However, quintuple or higher combined mutations were not recorded or were scarce [9, 15, 18, 42]. Herein, no sextuple or septuplet genotypes were noted in combined *dhfr–dhps*; however, the quintuple mutations were due to the combinations of IRNI–AGKAA and IRNI–FGKAA. These results highlight the fact that within the time of use, the number of haplotypes increases under the drug pressure. An in vitro study reported that the A_437_G mutant of *Pfdhps* exhibited a lesser degree of tolerance toward sulfadoxine than the double A_437_G–K_540_E mutant of *Pfdhps*. Therefore, quadrupleIRNI-G mutants exhibit a less noxious effect in parasites than quintuple mutants associated A_437_G–K_540_E mutations [58]. Herein, no mutations were detected at the 540 position (Table 1), and neither the quintupleIRNI–SGEAA nor the sextupleIRN**L**–SGEAA mutations were linked to SP drug failure (Table 2). These outcomes support the continued effectiveness of SP.

The emergence of artemisinin resistance has been reported in Myanmar, Vietnam, Laos, and China [19–21]. In fact, a wide range of *Pfk13*-propeller domain mutations linked to artemisinin resistance played a role in parasite clearance [19, 59]. However, A_578_**S** mutation was the most widespread in sub-Saharan Africa [52]. ACT is administered as the first-line treatment for non-severe malarial infection in Gabon from 2003 [47], and evidence of malarial morbidity decline in Libreville up to 2008 was reported following free access to and the huge distribution of ACT [31]. Among all the investigated samples, no mutations that have previously been linked to artemisinin resistance were noted. Furthermore, few non-synonymous mutations were identified in some areas of Gabon. The results of this study are consistent with previous findings [18, 45]. The current study included a small number of patients from one healthcare facility; thus, it may be necessary to expand the screening population to include patients from various health centers. This will potentially enhance the representative study scale of the situation of genetic polymorphisms associated with resistance to these drugs in the population of Libreville, Gabon.

## Conclusions

High frequencies of *Pfdhfr* and *Pfdhps* mutant haplotypes among the studies population were observed. The constant rise of mutations associated with SP drug resistance more than 20 years following its use for treating uncomplicated malaria cases that predated the implementation of IPTp–SP is alarming. However, despite several investigations, the haplotypes associated with drug failure are yet to be established. The constant increase of drugs resistant haplotypes sets across time and place over the country, constitute a permanent danger leading to drugs failure. Thus, to control the epidemiology accompanied by the administration of the treatment drugs, regular monitoring of drug efficacy must be mandated across the country.

## Supplementary information

**Additional file 1:  Table S1**. Primer pairs used for theprimary and secondary amplification of drug resistance genes.

## Data Availability

All data generated or analysed during this study are included in this published article.

## Abbreviations

ACT: Artemisinin-based combination therapy
AL: Artemether–lumefantrine
ASAQ: Artesunate–amodiaquine
DHFR: Dihydrofolate reductase
DHPS: Dihydropteroate synthase
IPTp: Intermittent preventive treatment in pregnancy
Pf k13: Plasmodium falciparum kelch 13
SP: Sulfadoxine–pyrimethamine
WHO: World Health Organization
