# Supplementary material for: Assessment of genetic polymorphisms associated with malaria antifolate resistance among the population of Libreville, Gabon
Source: Malar J. 2023 Jun 14;22:183. doi: 10.1186/s12936-023-04615-1 (PMC10265907; doi:10.1186/s12936-023-04615-1)
Supplement: Supplementary file 1 — Additional file 1: Table S1. Primer pairs used for theprimary and secondary amplification of drug resistance genes. [file 12936_2023_4615_MOESM1_ESM.docx]

**Additional file 1.** Primer pairs used for the primary and secondary amplification of drug resistance genes

| **Genes** | **Amplification** | **Primers sequence (5'–3')** | **References** |
| --- | --- | --- | --- |
| *Pfdhfr* | Primary | ACGTTTTCGATATTTATGC | [38] |
|  |  | TCACATTCATATGTACTATTTATTC |  |
|  | Secondary | ATGATGGAACAAGTCTGC | [37] |
|  |  | CTAGTATATACATCGCTAACA |  |
| *Pfdhps* | Primary | TTTTGTTGAACCTAAACGTG | [39] |
|  |  | AAACGTCATGAACTCTTATTAGAT |  |
|  | Secondary | TTCCTCATGTAATTCATCTGA | [37] |
|  |  | TTCCTCATGTAATTCATCTGA |  |
| *PfK13* | Primary | GGGAATCTGGTGGTAACAGC | [19] |
|  |  | CGGAGTGACCAAATCTGGGA |  |
|  | Secondary | GCCTTGTTGAAAGAAGCAGA |  |
|  |  | GCCAAGCTGCCATTCATTTG |  |
|  |  |  |  |
